# CHA2DS2-VASc Score as an Independent Predictor of Suboptimal Reperfusion and Short-Term Mortality after Primary PCI in Patients with Acute ST Segment Elevation Myocardial Infarction

**DOI:** 10.3390/medicina55020035

**Published:** 2019-02-01

**Authors:** Ammar Ashoori, Hamidreza Pourhosseini, Saeed Ghodsi, Mojtaba Salarifar, Ebrahim Nematipour, Mohammad Alidoosti, Ali-Mohammad Haji-Zeinali, Yones Nozari, Alireza Amirzadegan, Hassan Aghajani, Arash Jalali, Zahra Hosseini, Yaser Jenab, Babak Geraiely, Negar Omidi

**Affiliations:** 1 Tehran Heart Center, Tehran University of Medical Sciences, Tehran 1411713138, Iran; Dr_a_ashoori@yahoo.com (A.A.); dsaeedgh@gmail.com (S.G.); Mojtaba.salarifar@gmail.com (M.S.); dr_nematipour@tums.ac.ir (E.N.); salidoosti@hotmail.com (M.A.); ali_zeinali_cardio@yahoo.com (A.-M.H.-Z.); Y_nozari@yahoo.com (Y.N.); aamirzadegan@yahoo.com (A.A.); Aghajanih@tums.ac.ir (H.A.); arjalali@tums.ac.ir (A.J.); yasjenab@gmail.com (Y.J.); babak_geraiely@yahoo.com (B.G.); negar.omidi@gmail.com (N.O.); 2 Department of Cardiology, Imam Khomeini Hospital, Tehran University of Medical Sciences, Tehran 1411713138, Iran; Zmed702@yahoo.com

**Keywords:** no-reflow, STEMI, CHA2DS2-VASc score, reperfusion, mortality

## Abstract

*Background and objectives*: We aimed to demonstrate the clinical utility of CHA2DS2-VASc score in risk assessment of patients with STEMI regarding adverse clinical outcomes particularly no-reflow phenomenon. *Materials and Methods*: We designed a retrospective cohort study using the data of Tehran Heart Center registry for acute coronary syndrome. The study included 1331 consecutive patients with STEMI who underwent primary angioplasty. Patients were divided into two groups according to low and high CHA2DS2-VASc score. Angiographic results of reperfusion were inspected to evaluate the association of high CHA2DS2-VASc score and the likelihood of suboptimal TIMI flow. The secondary endpoint of the study was short-term in-hospital mortality of all cause. *Results*: The present study confirmed that CHA2DS2-VASc model enables us to determine the risk of no-reflow and all-cause in-hospital mortality independently. Odds ratios were 1.59 (1.30–2.25) and 1.60 (1.17–2.19), respectively. Moreover, BMI, high thrombus grade, and cardiogenic shock were predictors of failed reperfusion (odds were 1.07 (1.01–1.35), 1.59 (1.28–1.76), and 8.65 (3.76–24.46), respectively). We showed that using a cut off value of ≥ two in CHA2DS2-VASc model provides a sensitivity of 69.7% and specificity of 64.4% for discrimination of increased mortality hazards. Area under the curve: 0.72 with 95% CI (0.62–0.81). *Conclusions*: Calculation of CHA2DS2-VASc score applied as a simple risk stratification tool before primary PCI affords great predictive power. Furthermore, incremental values are obtained by using both CHA2DS2-VASc and no-reflow regarding mortality risk assessment.

## 1. Introduction

In the era of revascularization, primary percutaneous intervention (PCI) is the mainstay of the treatment of acute ST segment elevation myocardial infarction (STEMI) owing to favorable efficacy rather than that of thrombolysis. One of the leading complications associated with primary PCI is suboptimal reperfusion of the endangered myocardium in the territory of corresponding epicardial coronary artery. The no-reflow phenomenon occurs because of heterogeneous factors including distal embolization of debris pertaining to ulcerated atherosclerotic plaques, microvascular damage, vasospasm, insults of oxidative stress, and reperfusion injury. This entity involves about 5–15% of cases undergoing revascularization. Thus, we need a reliable risk stratification tool, which properly predicts the incidence of no-reflow regarding its multifactorial pathogenesis [1,2,3,4,5].

CHA2DS2 and CHA2DS2-VASc score models are widely applied to predict the risk of subsequent thromboembolic events in patients with atrial fibrillation [6]. Furthermore, such instruments have represented ample power in estimating major adverse cardiovascular outcomes in the setting of acute coronary syndrome. Therefore, we have assessed prognostic yield of this clinical score to estimate the risk of no-reflow following primary PCI and stenting.

## 2. Materials and Methods

We have accomplished a retrospective cohort study among 1331 consecutive patients with STEMI diagnosis who underwent primary PCI in Tehran Heart Center between February 2016 and March 2018. Patients were divided into two groups according to low and high CHA2DS2-VASc score. Then we compared these groups with respect to baseline characteristics. Angiographic results of reperfusion were inspected to evaluate the association of high CHA2DS2-VASc score and the likelihood of suboptimal TIMI flow. The secondary endpoint of the study was short-term in-hospital mortality of all cause. The median length of hospital stay determined as follow up period was six days. Coronary angiography was performed for all patients in addition to standard medical treatment of acute coronary syndrome. Thus, each participant received a loading dose of P2Y12 inhibitor (including 600 mg clopidogrel as the main agent and prasugrel 60 mg for a minority subgroup (1.2%)) accompanied with atorvastatin (80 mg), unfractionated heparin, and aspirin (300 mg). If use of a glycoprotein IIb/IIIa receptor antagonist was planned, 50-to 70-unit /kg IV bolus of unfractionated heparin to achieve therapeutic ACT (Activated clotting time) was administered. Otherwise, 70-to 100-unit /kg bolus dose of UFH to achieve a therapeutic ACT had been considered. Patients either with a delayed diagnosis of STEMI or deferred arrival whose symptoms persisted longer than 12 h were excluded. Furthermore, subjects who had non-significant stenosis in the culprit vessel or their coronary anatomy was not eligible to perform coronary angioplasty. Patients with favorable criteria for CABG without primary PCI, stenosis of the SVGs (saphenous vein grafts) as culprit lesions, and coronary artery dissection were also ruled out. Aspiration of thrombus particles and administration of glycoprotein IIb/IIIa inhibitor were determined regarding the clinical opinion of interventional cardiologist. STEMI diagnosis was verified according to a history of a typical chest pain accompanied with ST-segment elevation at the J point of 1 mm or more in at least two contiguous leads with the following cut points.

ST elevation ≥0.1 mV in all leads (except V2-V3). In leads, V2 and V3 these cut off values apply: ≥0.2 mV for men ≥40 years, ≥0.25 mV in men under 40 years, ≥0.15 mV in women. Furthermore, new left bundle branch block (LBBB) or meeting the Sgarbossa’s Criteria [1,7].

The TIMI flow was determined before and after primary PCI, comprised of four grades:

TIMI-0 indicates that there is no forward coronary flow in angiography beyond the site of stenosis or occlusion, TIMI-1 describes a poor distal antegrade flow leading to incomplete filling of the artery, TIMI-2 demonstrates a deferred slow frontward stream that fills the distal territory completely, TIMI-3 depicts normal coronary flow [8].

Myocardial blush grades (MBG) were quantified based on the previous classifications. Absence of myocardial blush or contrast density denotes Grade 0. Grade 1 refers to minimal contrast density. Grade 2 is known as observation of moderate myocardial blush but less than that obtained during an angiography of a contralateral or ipsilateral non-infarct-related coronary artery, and Grade 3 indicates “normal myocardial blush or contrast density comparable with that obtained during an angiography of a contralateral or ipsilateral non-infarct-related coronary artery” [9]. We reviewed angiography sine films in order to evaluate no-reflow/slow flow incidence using a combination of TIMI flow and MBG. Presence of TIMI flow <3 with any MBG grade or TIMI flow 3 accompanied with MBG 0 or 1 were considered as suboptimal reperfusion. Successful reperfusion was defined as TIMI flow 3 with MBG 2 or 3.

CHA2DS2-VASc risk score was computed for each subject based on the definition proposed by Lip et al. [6]. This risk assessment tool is a constellation subtending eight components with specified scores. The acronym represents as heart failure (C), hypertension (H), age ≥ 75 years (A2), diabetes mellitus (D), stroke (S2), vascular disease (V), age 65 to 74 years (A), and female gender (as a sex category [Sc]). Quantified values pertaining to stroke and Age over 75 are determined with two points while 1 point was assigned to each of the remaining variables [6].

Thrombus burden of the target lesions were quantified based on the classification of Gibson et al. [10].

Thus, an angiographic appraisal of the thrombus content with respect to the relative atherosclerotic structure dimensions, providing a simple score ranging from Grade 0 (absence of thrombus) to Grade 5 (very large thrombus leading to near-total occlusion). Besides, the thrombus grade variable was further stratified into high (Grade > 2) and low thrombus load.

ACC-AHA Task Force Definition, according to Ellis et al., was applied to ascertain the coronary lesion complexity types as A, B1, B2, and C [11]. A creatinine clearance under 60 mL/min/1.73 m^2^, calculated by Cockroft equation accompanied with previous history of renal failure and sonographic characteristics was defined as chronic kidney disease [12]. We defined cardiogenic shock as a systolic blood pressure of less than 90 mm Hg or mean arterial pressure under 70 accompanied with impaired organ perfusion. 

The present study complied with the principles of the Declaration of Helsinki and the ethics committee of Tehran University of Medical Sciences approved the investigation design. The local code for Tehran Heart Center ethics approval was 6432-A. 

## 3. Statistical Analysis

Continuous variables were expressed as mean ± standard deviation while categorical variables were shown by percentages. Two-tailed Student’s *t*-test and Mann–Whitney U test were recruited to compare continuous variables with and without normal distribution, respectively. Chi-square test was used to show the difference of categorical variables. We have also demonstrated isolated and complex interaction-mediated impacts of predictors of no-reflow phenomenon. Therefore, univariate and multivariate regression analyses were performed to evaluate unadjusted and adjusted association of potential risk factors specially CHA2DS2-VASc and suboptimal coronary flow. We determined the predictive utility of the CHA2DS2-VASc score for subsequent outcomes including no-reflow and in-hospital mortality using receiver operating characteristic curves. Statistical significance was confirmed with a *p*-value < 0.05. All analyses were conducted using SPSS version 22 (SPSS Inc., Chicago, IL, USA).

## 4. Results

In total, 1331 eligible patients in the Tehran Heart Center registry of STEMI were evaluated for CHA2DS2-VASc score and evidence of no-reflow by reviewing final angiographic results of the primary PCI procedure. Mean age of the study population was 59.41 ± 11.78 years and the average global EF (ejection fraction) was 41.52 ± 7.4. Majority of the subjects were male (0.80). Total frequency of no-reflow was 5.6% (*n* = 74). The most prevalent culprit vessel was LAD (left anterior descending artery) with 48.5% (versus left circumflex (LCX) and right coronary artery (RCA) with 11.6 and 29.3%, respectively). PCI of ramus vessel, diagonal artery, posterior descending artery, posterolateral branch (PLB), and obtuse marginal branch (OM), accounted nearly 10.6%, altogether. These frequencies were also similar in subgroups of low and high CHA2DS2-VASc score. Frequency of no-reflow phenomenon was 5.6% which was predominantly greater among high CHA2DS2-VASc subjects (7.1% vs. 5.1%, *p*: 0.03). Total in-hospital mortality incidence rate was 0.025. Mortality rates of high-risk participants (CHA2DS2-VASc > 2) was significantly higher than that of low risk ones (6.8% vs. 1.4%, *p*: 0.012). GPIIbIIIa inhibitor agents were used in 74.9% of patients while only one subject had received fibrinolytic therapy (streptokinase) prior to PCI.

Table 1 represents Baseline characteristics of the participants with respect to the primary risk profiles. Therefore, individuals with low CHA2DS2-VASc score were compared with high-risk category (<3 vs. ≥3). We observed that some of substantial variables, which may potentially contribute to the no-reflow occurrence, were identical in both groups. These parameters were initial TIMI flow, thrombus grade, thrombosuction, length of stents, complexity of lesions (based on ACC-AHA classification), and anatomic segments in which angioplasty was done (proximal or distal and target territory). On the other hand, GPIIbIIIa inhibitors were used in a lower proportion of patients with high CHA2DS2-VASc score (76.7% vs. 68.9%). However, we have adjusted the influence of potential interactions for several predictors of suboptimal flow. As expected, the prevalence of diabetes mellitus (DM) and hypertension (HTN) were significantly greater in high CHA2DS2-VASc group. The frequencies of DM and HTN were (71.2% vs. 27.1%) and (87.5% vs. 33.0%) respectively. Peripheral vascular diseases were observed among 4.21% of low risk and 5.4% of high-risk group (*p*: 0.67). The Median symptom-to balloon times (interquartile ranges in minutes) were similar for both groups. Median times were calculated as 146 (114–235) against 138 (98–241) for low and high-risk group respectively (*p*-value: 0.085). Mean glomerular filtration rate (GFR) was greater among patients with low CHA2DS2-VASc score ((116.8 ± 19.6 vs. 86.6 ± 24.5) *p*-value: 0.041). Similar patterns regarding the use of medications prior to STEMI events were observed between the two groups except for oral hypoglycemic agents and insulin (more common in high CHA2DS2-VASc group due to higher prevalence of DM). The prevalence of pharmacotherapy with statins, ACEI (Angiotensin converting enzyme inhibitors), ARBs (Angiotensin receptor blockers), and beta blockers were (36.2% vs. 32.7%), (25.4% vs. 29.8%), (20.95 vs. 18.6%), (15.8% vs. 19.5%), respectively. All *p*-values pertaining to drug history of the subjects were above 0.05.

Univariate impacts of stent length, stent diameter, and glomerular filtration rate on no-reflow were 1.012 (0.98–1.03), 0.95 (0.68–2.67), and 1.26 (0.84–1.88) respectively. On the contrary, thrombus grade, BMI, and cardiogenic shock had significant univariate associations with no-reflow phenomenon. Corresponding odds ratios were (3.21 (1.99–5.18) *p*: 0.001), (1.06 (1.04–1.46) *p*: 0.02), and (14.30 (3.75–54.46) *p*: 0.001) respectively. Administration of GpIIbIIIa was associated with a lower probability of no-reflow in univariate tests (0.507 (0.26–0.97) *p*: 0.03) but this effect was disappeared in multivariable regression analysis (0.69 (0.34–1.42) *p*: 0.32). 

Table 2 demonstrates the integrated multivariate and purified effects of these variables on incidence of failed reperfusion. Hence, a significant association was found between CHA2DS2-VASc and final suboptimal flow (odds ratio: 1.59 (1.30–2.25).

**Figure 1:**
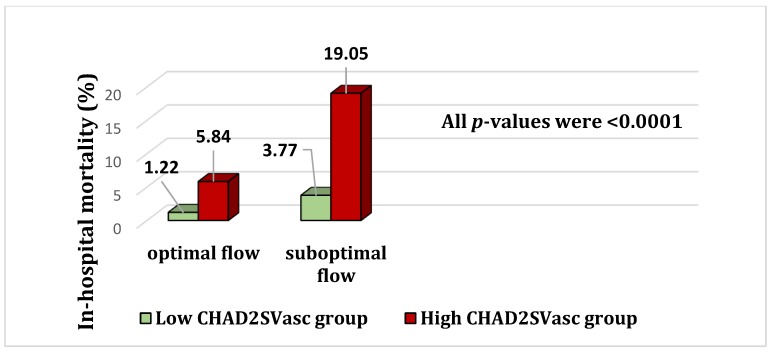
In hospital short -term mortality of patients following primary PCI regarding final TIMI flow and initial CHADS2VASc score. Green bars depute low CHADS2VASc group (<3) while red bars represent high CHADS2VASc category (≥3).

**Figure 2:**
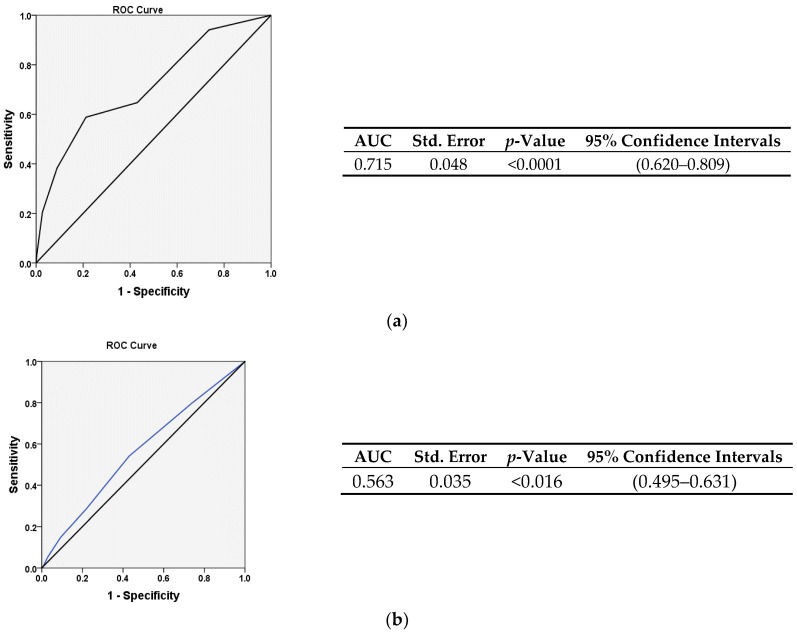
(**a**) AUC (Area under the curve) of CHAD2Svasc for Discrimination of Mortality. prediction of in-hospital short-term mortality of patients following primary PCI using CHADS2VASC tool. (**b**) AUC (Area under the curve) of CHAD2Svasc for Discrimination of no-Reflow. Predictive value of CHADS2VASc score for suboptimal TIMI flow.

Table 3 reveals considerable independent utility of CHA2DS2-VASc score to predict short-term mortality. In addition, we have shown the incremental value of CHA2DS2-VASc model to no-reflow in predicting mortality via a subgroup analysis (Figure 1). In Figure 2A,B, AUC of ROC graphs illustrate the power of CHA2DS2-VASc score in prediction of mortality and no-reflow phenomenon, respectively.

Determining CHA2DS2-VASc score > 2 as a predictor of in-hospital mortality seems to be an appropriate cut-off value owing to a sensitivity of 69.7% and a specificity of 64.4% (Figure 2).

When we assessed the isolated effects of CHA2DS2-VASc constellation, heart failure and hypertension were associated with no-reflow. Multivariate odds were 1.68 (1.09–4.32) and 1.94 (1.34–7.54) respectively. However, diabetes mellitus showed a trend toward borderline significance (1.58 (0.98–2.52), *p*: 0.055). Furthermore, older age (above 75) and heart failure were related to mortality, significantly. Adjusted odds were (1.83 (1.03–3.45), *p*-value: 021) and (2.56 (1.32–6.18) *p*-value: 0.001) respectively.

## 5. Discussion

Results of the present study verified the clinical implication of CHA2DS2-VASc model playing an extra role in prediction of adverse outcomes following primary PCI. We found that using this risk score is able to discriminate STEMI patients at higher risk of no-reflow after angioplasty. Furthermore, our findings demonstrated that increased CHA2DS2-VASc score is also an independent predictor of in-hospital mortality rather than a surrogate measure only. Thus, we may need to focus on management of its components. Although this scoring tool was primarily developed and validated to aid prediction of ischemic stroke among patients with atrial fibrillation, additional utilities were introduced thereafter [6,13]. There are multiple risk stratification models for assessment of prognosis after acute coronary syndrome. However, some models are complex or contain variables, which are not available at presentation data. Due to time limits determined for revascularization of STEMI, practical benefits of a simple accustomed risk score such as CHA2DS2-VASc gets more prominent [14,15]. Herein, our findings revealed a dual prognostic utility of CHA2DS2-VASc for both suboptimal reperfusion and short-term mortality. 

Failure of reperfusion so called no-reflow often correlates with extended myocardial necrosis and poor clinical outcome irrespective of infarct size [16,17].

Pathophysiologic mechanisms recognized for no-reflow elucidate the pathways of prediction and management, in part. Initial cornerstone of suboptimal perfusion is a severe ischemic event accompanied with endothelial cell necrosis and dysfunction, microvascular obstruction with necrotic debris, distal thromboembolism. Reperfusion injury following primary PCI, extravascular fluid accumulation and luminal narrowing, autonomic vasoconstriction, diminished nitric oxide production; oxygen-free radicals are also among substantial contributing factors. Then, vasoconstriction due to endothelial dysregulation and platelet activation cascade takes place. Cytotoxic signals recruit inflammatory cells and immune response during the next phase, which in turn results in additional damage (apoptosis), and fibroblast infiltration. Replacement fibrosis, scarring, poor myocardial healing, adverse remodeling, LV dysfunction, and infarct size expansion occur ultimately [18]. Although optimal coronary flow is resumed in about 50% of patients, both short- and long-term mortality rates increase invariably. For example, Ndrepepa G. et al. have demonstrated that no-reflow elevated one-year adjusted risk of death after primary PCI by 3-folds [17]. Thus, early identification of STEMI patients vulnerable to no-reflow is essential to prevent or minimize the risk of unsuccessful reperfusion. Diabetes mellitus, as a constituent of CHA2DS2-VASc score impairs normal endothelial function and perpetuates ischemic reperfusion injury [18,19,20]. Similar adverse effects are delivered in hyperlipidemia. Therefore, adherence to intensive statin treatment as well as optimal plasma glucose management prepare the microvasculature to prevent or attenuate no-reflow phenomenon. Dyslipidemia is also associated with both cerebrovascular and cardiovascular disorders, which are considered in CHA2DS2-VASc model [20,21]. Furthermore, high blood pressure, female gender, acute and chronic kidney injury, high levels of inflammatory biomarkers, involvement of LAD territory, and complex atherosclerotic plaques containing high thrombus burden are of great significance. Rezkalla S.H. et al. have suggested decreasing door to balloon time, appropriate hypertension control in addition to avoiding hyperglycemia and hypercholesterolemia. True discrimination of patients who are susceptible to no-reflow causes using tailored strategies to improve final coronary perfusion. In this context, applying prophylactic intracoronary dilators, primary stenting, short stents with adjusted lower pressure, thrombus aspiration in particular cases, and occasionally distal protection devices may be beneficial [21,22,23,24]. A critical point is the decision about whether the case is feasible for deferred stenting method or not. According to the DEFER-STEMI trial, late stenting in particular pre-specified subjects undergoing primary angioplasty improves myocardial salvage index and reduces suboptimal reperfusion rate [25]. In this regard, CHA2DS2-VASc model affords a simple, time saving tool for risk stratification.

Overlapping of risk factors and shared pathophysiologic pathways can interpret the clinical utility of CHA2DS2-VASc score in prediction of both stroke and ischemic events such as no-reflow. Among these factors are microvascular dysfunction, atherothrombosis, and embolization. Simultaneously all of the components including diabetes, hypertension and heart failure predict major adverse clinical events in ACS. Hence, in line with previous studies, it was not surprising to observe a considerable association of the model with in-hospital mortality [26,27,28]. However, the incremental value of adding CHA2DS2-VASc score to no-reflow was worth noting. In contrast with majority of the reports, only two components including hypertension and heart failure showed significant correlation with no-reflow. We found greater in-hospital mortality risk in STEMI patients older than 75 and among who had heart failure. Previous studies demonstrated established association of age, and reduced EF with MACE of ACS patients. However, we did not observe previously known impacts of female gender, concomitant cerebrovascular and peripheral arterial disorders on mortality or no-reflow. Most of these studies have confirmed enhanced mortality and morbidity in the presence of PAD but there is not enough evidence supporting the association of PAD and no-reflow. Likewise, few studies displayed independent predictive role of diabetes mellitus (not only hyperglycemia), which is similar to our results. However, we found a borderline statistical significance for this relationship. Overall, the question about whether high CHA2DS2-VASc has an independent additional effect beyond its components remains controversial [29,30,31].

Previous investigations have detected various determinants of suboptimal reperfusion following primary PCI. Heart failure (embedded in CHA2DS2-VASc score), cardiogenic shock, and high thrombus burden were found in the present study in line with former reports. Association of body mass index (BMI) with no-reflow that remained persistent after multiple adjustments was new in our study. Similar to our results, Mirbolouk et al. and Ipek et al. concluded that stent length over 20 mm, stent diameter, hyperlipidemia, declined GFR, history of CKD, thrombusuction, and smoking did not affect the endpoint. Use of tirofiban was not associated with decreased no-reflow rate, which was concordant with our findings. However, we used different inhibitors of glycoprotein IIbIIa. Ipek et al. excluded patients who had received these agents (mainly eptifibatide). In contrast with present study, Mirbolouk et al. demonstrated that initial TIMI flow greater than 1 correlates with lower likelihood of final suboptimal flow (OR: 0.06 (0.02–0.20)). BAYRAMOĞLU et al. declared the impacts of long stent (>20 mm) and thrombus grade on no-reflow risk. Odds ratios 3.607 (1.932–6.734), and 3.139 (1.081–9.113), respectively. We showed significant association of high thrombus burden with no-reflow incidence, However, further adjustments blunted this effect. We did not observe beneficial effect of thrombus aspiration and glycoprotein IIbIIIa inhibitor (in multivariate analysis) which was concordant with literature. 

In conclusion, traditional CHA2DS2-VASc model is a useful easily available risk assessment tool to stratify STEMI patients who are more prone to no-reflow and failed reperfusion. Besides, we have a useful surrogate measure of in-hospital mortality independent of no-reflow [19,25,32,33,34,35,36,37,38].

## 6. Study Limitations

Our study had several limitations. First, the retrospective design based on a single center registry data. The incidence of no-reflow in our study was low. Hence, if a greater number of events were observed we would be able to show the true strength of the associations.

Angiographic assessment of thrombus burden is less sensitive and less specific than intravascular ultrasound or optical coherence tomography. Furthermore, measurements of ejection fraction and blood pressure are influenced in acute setting of myocardial infarction. Acidosis–alkalosis state, blood gas mixture, dosage and types of anticoagulant, protocol of balloon dilation, and pain time to balloon interval were not considered in our study. Angiographic (visual) grading of no-reflow is prone to a large degree of bias and both intra-observer and inter-observer reliability bias. Only one observer determined the visual grading results (TIMI and MBG) in our investigation. However, he was blinded to the classifications and assignments of the patients to each category of the study. A solution for this potential bias is using a computer-based scaling system to measure these parameters. 

## Figures and Tables

**Table 1:** Baseline characteristics of participants among low risk and high-risk patients according to CHA2DS2-VASc score.

Variables		CHA2DS2-VASc Score Category		*p*-Value
		Low (<3) *n* = 1036	High (≥3) *n* = 295	
Sex	Female	97 (9.4%)	169 (57.3%)	**<0.0001**
	Male	939 (90.6%)	126 (42.7%)	**<0.0001**
Initial TIMI flow	0	566 (54.6%)	171 (58%)	0.337
	1	154 (14.9%)	32 (10.8%)	0.316
	2	222 (21.4%)	67 (22.7%)	0.640
	3	94 (9.1%)	25 (8.5%)	0.515
History of CKD		8 (0.8%)	22 (7.5%)	**<0.0001**
Previous CABG		12 (1.2%)	11 (3.7%)	**0.005**
Dyslipidemia		500 (48.35)	165 (55.9%)	**0.021**
smoking		466 (45%)	34 (11.5%)	**<0.0001**
GP IIb-IIIa-inhibitor		795 (76.7%)	203 (68.9%)	**0.003**
Cardiogenic shock		7 (0.71%)	2 (0.69)	0.897
ACC/AHA classification for complexity of the lesions	B1	67 (6.5%)	20 (6.8%)	0.975
	B2	172 (16.6%)	48 (16.3%)	0.966
	C	796 (76.8%)	228 (76.9%)	0.945
PCI location	Non-proximal	540 (52.1%)	156 (52.9%)	0.495
	ostial	122 (11.8%)	41 (13.9%)	0.499
	proximal	374 (36.1%)	98 (33.2%)	0.55
High Thrombus grade		325 (31.4%)	97 (32.9%)	0.62
Thrombusuction		10.4%	9.9%	0.913
Age		56.09 ± 10.16	71.15 ± 9.35	**<0.0001**
Stent length		27.47 ± 9.39	28.09 ± 9.55	0.334
Stent diameter		3.03 ± 0.45	2.84 ± 0.40	**0.01**
Creatinine		0.94 ± 0.35	1.07 ± 0.61	**0.01**
Ejection Fraction		42.66 ± 9.72	37.90 ± 11.69	**<0.0001**
BMI		27.54 ± 4.16	28.27 ± 4.53	**0.011**

**Table 2:** Multivariate regression analysis of the association between CHA2DS2-VASc score and no-reflow phenomenon.

Predictors	OR1 (95% CI)	Sig	OR2 (95% CI)	Sig	OR3 (95% CI)	Sig
CHA2DS2-VASc score	1.34 (1.09–1.64)	0.005	1.52 (1.01–2.10)	0.012	1.59 (1.30–2.25)	0.008
BMI	1.07(1.01–1.35)	0.032	1.11 (1.01–1.22)	0.042	1.12 (1.01–1.24)	0.033
Thrombus grade (high vs. low)	1.59 (1.28–1.76)	0.002	1.66 (0.57–4.90)	0.36	1.67(0.56–4.99)	0.34
Cardiogenic shock	8.65(3.76-24.46)	<0.0001	6.34 (2.15–15.56)	<0.0001	3.25(1.23–0.8.63)	<0.0001

OR (95%CI): Odds Ratio (95% Confidence Interval), Sig: statistical significance. BMI: Body Mass Index.OR1: Odds ratio values were adjusted for smoking, initial TIMI flow, stent length, and stent diameter. OR2: adjustments were done for variables applied in OR1 plus creatinine (GFR), global EF (ejection fraction), PCI time (minutes), PCI location (ostial, proximal, and non-proximal). AHA/ACC classification of lesions, thrombusuction, and, use of GPIIbIIIa inhibitor, hyperlipidemia, and history of cerebrovascular events.OR3: We performed adjustments for variables included in OR2 in addition to the coronary territory of culprit lesion including left main, left anterior descending, left circumflex or right coronary artery.

**Table 3:** Multivariate regression analysis of the association between CHA2DS2-VASc score and short-term in-hospital mortality of STEMI patients.

Predictors	Univariate (95% CI)	*p*-Value	Multivariate (95% CI)	*p*-Value
CHA2DS2-VASc score	1.82 (1.45–2.26)	<0.0001	1.60 (1.17–2.19)	0.004
No-Reflow	3.87 (1.55–9.67)	0.004	5.33 (1.65–17.20)	0.005
Thrombus grade (high vs. low)	2.81 (1.41–5.59)	0.003	2.71 (1.20–7.23)	0.041
Creatinine clearance (<60 vs. ≥60)	2.48 (1.62–3.80)	<0.0001	2.12 (1.41–3.19)	<0.0001

Multivariate adjustments were done for age, sex, initial TIMI flow, smoking, PCI coronary territory, hemodynamic status (cardiogenic shock or stable condition), stent diameter, LV ejection fraction (heart failure), and use of GPIIbIIIa, dyslipidemia, and BMI. Creatinine clearance expressed in mL/min/1.73 m^2^.
